# Phase transformation in tungsten oxide nanoplates as a function of post-annealing temperature and its electrochemical influence on energy storage†

**DOI:** 10.1039/d0na00423e

**Published:** 2020-08-06

**Authors:** Shobhnath P. Gupta, Harishchandra H. Nishad, Sanjay D. Chakane, Suresh W. Gosavi, Dattatray J. Late, Pravin S. Walke

**Affiliations:** National Centre for Nanoscience's and Nanotechnology, University of Mumbai Mumbai-400098 India pravin.w@nano.mu.ac.in shivshripsw@gmail.com +91 8380832183; Department of Physics, Arts, Science and Commerce College Indapur, Affiliated to Savitribai Phule Pune University Pune-413106 India; Department of Physics, Savitribai Phule Pune University Pune-411007 India; Centre for Nanoscience and Nanotechnology, Amity University Mumbai-410206 India

## Abstract

The morphology and crystal structure of electrode materials have an enormous impact on their electrochemical properties for employment in supercapacitors for various applications. In this study, the transformations of the crystal structure of WO_3_·H_2_O nanoplates were conducted by post-annealing at 200 °C and 400 °C. The morphological and structural evolution of the electrodes was studied *via* FEG-SEM, HRTEM, FTIR, XRD, and Raman spectroscopy. The phase transition and enhanced degree of crystallinity were observed with increasing temperature. The orthorhombic structures of the hydrate WO_3_·H_2_O (W80), the mixed-phase with mesoporous structure (W200), and finally the monoclinic phase of WO_3_ structures (W400) were achieved at annealing temperatures of 80 °C, 200 °C, and 400 °C respectively. The electrochemical performance of electrode W200 showed the highest specific capacitance of 606 F g^−1^ as compared to electrode W80 (361 F g^−1^), and was two-fold greater than electrode W400 (302 F g^−1^) at a current density of 1 A g^−1^. Moreover, electrode W200 exhibited excellent cyclic stability of 89% at an ultrahigh scan rate of 100 mV s^−1^ after 4000 cycles. The results highlight that the mixed-phase WO_3_ nanoplates would make a suitable electrode material for supercapacitors with desired electrochemical features.

## Abstract 2

The schematic representation exhibits phase transformation of WO_3_ nanoplates at various annealing temperatures confirmed by XRD and Raman spectra. The superior electrochemical performance is achieved by the mixed phase WO_3_ crystal structure.

## Graphical abstract

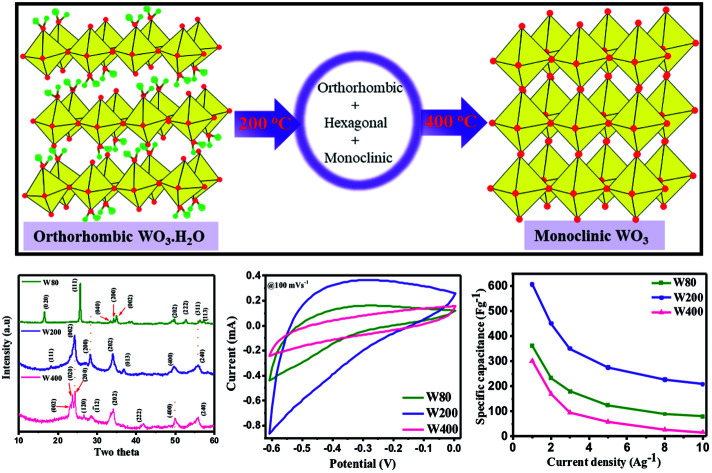

## 1. Introduction

The supercapacitor is a promising electrochemical energy storage device as compared to the battery, owing to its superior power density, rapid charge–discharge rate, and excellent life cycle.^1–4^ Due to the kinetics involved in charge storage across the interface of the electrode and electrolyte, the supercapacitor can be categorized as an electrical double-layer capacitor (EDLC) and pseudocapacitor. In the EDLC charge storage mechanism, the ions are electro-statistically adsorbing at the interface between the electrode and electrolyte. The pseudocapacitor charge storage mechanism can arise due to three major processes as follows. (1) Underpotential deposition, where metal ions or protons are adsorbed on a metal surface; (2) redox pseudocapacitance, where faradaic redox reactions arise on the surface or near the surface of electrode materials; (3) intercalated pseudocapacitance, where there is the fast faradaic insertion of ions into the tunnel or inter-layers of the electrode material.^1,4^ The supercapacitor performance is highly governed by the physical and chemical properties of the electrode materials, including porosity, chemical composition, chemical, and thermal stability, surface modification, electrical conductivity, crystal structure, and phase stability of the resulting structure.^5–7^ Transition metal oxides (TMO), such as MnO_2_, RuO_2_, Fe_2_O_3_, NiO, Co_3_O_4_, MoO_3_, are highly promising electrode materials due to their various active sites for redox reactions as compared to carbon-based materials, developing non-faradaic processes *via* the electric double layer.^8–13^ Nevertheless, the TMOs seriously suffer from low conductivity, high toxicity as well as high cost.^14^ To solve these issues, tungsten oxide (WO_3_) is preferred as a cheap and abundant material, which has also found superior applications in antibacterial, electrochromic, photocatalysis, gas sensor, and field emission applications.^15–19^ It has received significant attention as an electrode material due to its various oxidation states (W^2+^ to W^6+^), and high-density (>7 g cm^−3^) for electrochemical energy storage devices.^20,21^ Furthermore, its various crystal phases, including monoclinic, tetragonal, orthorhombic, cubic, and triclinic, may form hexagonal, pentagonal, trigonal tunnel, and cavities.^22^ Among these various structures, the hexagonal structure with a large tunnel size of 0.6 nm is favorable for electrochemical ionic intercalation.^23^ These polymorphic phases are highly sensitive to temperature changes and have attracted significant attention for their electrochemical charge storage performance.^24^

Recently, Mitchell reported the monoclinic WO_3_·2H_2_O, orthorhombic WO_3_·H_2_O, and monoclinic γ-WO_3_ structure at room temperature, 120 °C and 350 °C, respectively.^25^ The γ-WO_3_ structure demonstrated the highest capacity of 420 C g^−1^ as compared to the monoclinic WO_3_·2H_2_O (360 C g^−1^) and orthorhombic WO_3_·H_2_O (386 C g^−1^). Augustyn *et al.* observed threefold increments in the capacitance of γ-WO_3_ (227 F g^−1^) as compared to monoclinic WO_3_·2H_2_O (75 F g^−1^).^26^ Hexing Li *et al.* observed a significant loss of capacitance after the annealing of h-WO_3_·H_2_O at 450 °C, which was attributed to the structural transformation from hexagonal to monoclinic WO_3_.^27^ Zongming Shao *et al.*, reported the capacitance of 391 F g^−1^ for h-WO_3_·0.28H_2_O, however, after annealing at 500 °C, the capacitance drastically decreased to 42 F g^−1^ with the formation of the monoclinic WO_3_ structure.^28^ Shim Joo *et al.*, obtained the as-prepared orthorhombic WO_3_·0.33H_2_O nanorods, which were further transformed into the hexagonal and monoclinic structures *via* post-annealing at 400 °C and 500 °C, respectively.^29^ These monoclinic WO_3_ nanorods demonstrated the highest photocurrent density of 2.26 mA cm^−2^ at 1.23 V. Thus, it is important to understand the fundamental characteristics of the phase transformations of the WO_3_ nanostructure, as its various applications demand annealing and/or higher temperature operations.

A porous network of electrode materials is highly remarkable for energy storage and conversion, which ensure abundant active sites for redox reactions and provide plentiful voids to accommodate ions during the charge–discharge process.^30–32^ The template-assisted, chemical etching and pyrolysis at high temperatures are typical preparation methods for forming porous structures.^33^ The template-assisted method is highly suitable for creating pores in the bulk materials using numerous templates such as anodic aluminum oxide (AAO) and silicate-based periodic mesoporous templates, namely, MCM-41, MCM-48, HMS, SBA-15, and so on.^34,35^ However it is difficult to create the pores in metal oxide nanosheets, and significant efforts are required to remove the template, such as the etching method. Practically, a pyrolysis method would be an easier and simpler approach to converting metal hydroxide sheets into the metal oxide; it significantly collapses the morphology and improves the structure architecture.

In this work, we report the preparation of the orthorhombic phase of the hydrate WO_3_·H_2_O nanoplate annealed at 80 °C (W80). Further annealing at 200 °C (W200) and 400 °C (W400) resulted in the phase transition to the tri-phase of the orthorhombic/hexagonal/monoclinic (W200) and pure monoclinic WO_3_ nanostructures (W400), respectively. The FESEM and HRTEM images revealed the creation of the pore structure on the W200 and W400 samples. The electrochemical performances of all samples were investigated using cyclic voltammetry and a charging/discharging process. The fundamental charge storage kinematics was explored by scan-rate-dependent electrochemical analysis and electrochemical impedance spectroscopy. The importance of crystal structure engineering in enhancing the electrochemical performance of the hydrate WO_3_·H_2_O nanoplate was systematically examined for suitable electrode materials of supercapacitors.

## 2. Experimental section

### 2.1 Materials and reagents

Sodium tungstate dihydrate (Na_2_WO_4_·2H_2_O), concentrated sulfuric acid (H_2_SO_4_), and ethanol were purchased from SD Fine, India. Nafion was purchased from Alfa Aesar, India. All the aqueous solutions were prepared using double distilled water (DDW).

### 2.2 Material synthesis

#### Synthesis of WO_3_·H_2_O nanoplates

The WO_3_·H_2_O nanoplates were prepared by a cost-effective, simple wet chemical method: 100 ml of aqueous 1 M H_2_SO_4_ solution was prepared and 0.5 g of Na_2_WO_4_·2H_2_O was slowly added with constant stirring at 100 RPM. Stirring was continued for 30 min more at room temperature (RT). Afterward, the solution was heated at 110 °C for 1 h and then naturally cooled down to RT. The obtained precipitate was washed thoroughly with DDW and finally with ethanol. The preparation of the WO_3_·H_2_O nanoplates can be represented by reaction (a) as follows:

a Na_2_WO_4_ + H_2_SO_4_ → WO_3_·H_2_O + Na_2_SO_4_

The powder was post-annealed at 80 °C, 200 °C and 400 °C for 4 h to obtain samples W80, W200 and W400, respectively.

### 2.3 Materials characterization

An X-ray diffractometer (Bruker D2 Phaser) with CuKα radiation and *λ* = 1.54056 Å in the 2*θ* range of 10–80 was used to study the crystal structure. Raman Microscopy (Model RENISHAW inVia, laser-multiline argon ion laser (514/488 nm); Raman spectral range of 100 cm^−1^ to 4000 cm^−1^) was used to examine the purity and for structural analysis. Fourier transform infrared spectroscopy (FTIR) (Varian 660-IR) was used to identify the functional groups present on the surface of the samples. A field emission scanning electron microscope (FESEM), (FEG Inspect 50, FEI) and a high-resolution transmission electron microscope (HR-TEM) with selected area electron diffraction (SAED), (TECNAI G2-20-TWIN TEM operating at 300 kV) were used for morphological and structural investigations. Surface area and pore size were investigated by nitrogen adsorption–desorption at 77 K using the Quantachrome Autosorb 1C.

### 2.4 Electrochemical measurements

The electrochemical measurements were carried out in a three-electrode setup using the PGSTAT302N Autolab system. A glassy carbon electrode, Hg/HgCl, and platinum wire were used as the working electrode, a reference electrode, and counter electrode, respectively.

#### Working electrode preparation for the three-electrode set up

The working electrode was prepared by the drop-casting method: 5 mg of active material was added to 2 ml ethanol–water (1 : 1 ratio) with 10 μl Nafion as a binder and sonicated for 10 min. The 10 μl solution was loaded on the working electrode and further dried for 2 h under the IR lamp (200 W). The mass loading of 0.36 mg cm^−2^ was maintained and aqueous 1 M H_2_SO_4_ solution was used as the electrolyte for all electrochemical measurements.

## 3. Results and discussion

### 3.1 Structural and morphological investigations

**Fig. 1:**
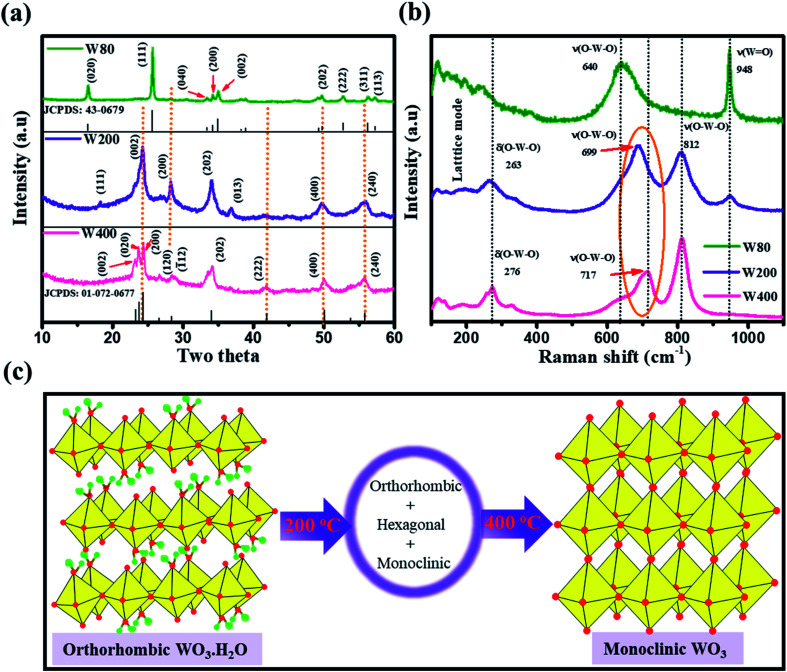
(a) XRD spectra and (b) Raman spectra of W80, W200, and W400 samples; (c) schematic representation of the crystal structure transformation as a function of annealing temperatures.

**Fig. 2:**
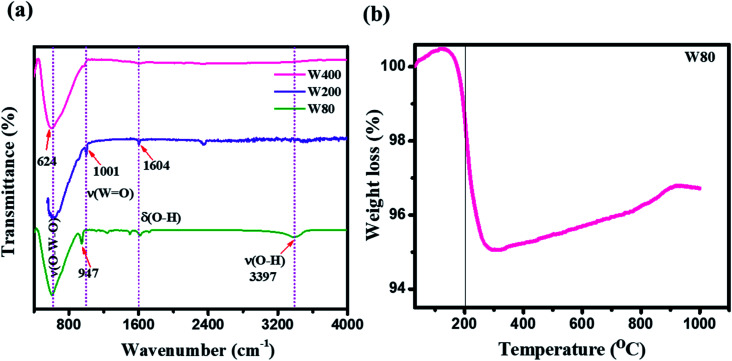
(a) FTIR spectra of W80, W200, and W400. (b) TGA curve of the W80.

The XRD measurements (Fig. 1(a)) illustrated the phase transition and crystal structure of the W80, W200, and W400 samples. The XRD spectrum of W80 showed the high-quality orthorhombic phase of the hydrate WO_3_·H_2_O structure with lattice constants *a* = 5.249, *b* = 10.711, and *c* = 5.1333 (JCPDS: 43-0679).^36^ This structure is composed of a highly distorted WO_5_(OH)_2_ octahedron coordinated with five oxygen atoms and one water molecule (Fig. S1†). The WO_5_(OH)_2_ polyhedron is held together by strong covalent bonds *via* a bridging O–W–O network along the *ab*-plane, and remaining terminal oxygen atoms, and other axial water molecules along the *c*-axis. It was impossible to make a bond with the nearest sheets due to the hydrate molecules, thus leading to a layered structure. These hydrate molecules were ordered and confined between the layers and stabilized the structure. The XRD pattern of W200 represents the combination of three phases. Plane (111) at an angle of 18.38° corresponds to the orthorhombic phase (WO_3_·0.33H_2_O) (JCPDS: 35-0270); the highly intense plane (002) at 24.14° characterizes the hexagonal structure (h-WO_3_) (JCPDS: 85-2460), and the remaining peaks above 24.14° illustrate the existence of the monoclinic phase.^29,37–39^ On further annealing at 400 °C, W400 exhibited a pure monoclinic phase with lattice constants *a* = 7.306, *b* = 7.540, and *c* = 7.69 (JCPDS: 01-072-0677). The diffraction peak of W200 along the (111) plane completely vanished and the plane (002) corresponding to the hexagonal plane became a triplet, with the (002), (020) and (200) planes at angles of 23.14°, 23.68°, and 24.37° respectively. Thus, annealing at 400 °C resulted in the removal of the confined, ordered water molecules between the layers and formed a strong bridging covalent bond (O–W–O) along the *c*-axis, realizing a 3D monoclinic WO_3_ structure (Fig. S2†). The crystalline sizes (*D*) of W80, W200 and W400 were estimated by the Scherrer eqn (1) as follows:1
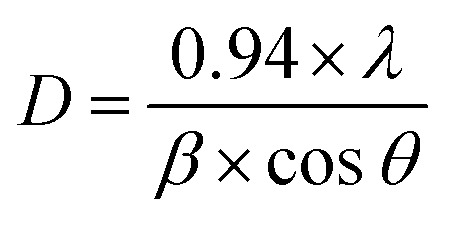
where *λ* is 1.54056 Å and 0.94 is a constant for the CuKα radiation, *β* is line broadening in the radian (FWHM). The obtained crystalline sizes of W80, W200 and W400 were 36.4 nm, 11.3 nm and 27.2 nm, respectively. Raman spectroscopy (Fig. 1(b)) is a powerful tool for distinguishing the crystalline symmetry and microstructure variations. The Raman spectra of the WO_3_ structure depicted the three major characteristic regions between 600 cm^−1^ to 900 cm^−1^, 400 cm^−1^ to 600 cm^−1^ and below 200 cm^−1^, which correspond to the stretching mode, deformation and lattice modes, respectively.^40^ The Raman spectrum of W80 exhibited the characteristic band of the terminal oxygen atom (W=O) at 948.3 cm^−1^. This peak strongly indicates the presence of confined hydrated molecules in the layered structure. However, the band at 640.1 cm^−1^ represents the stretching mode of the bridging *ν*(O–W–O) bond along the *ab*-plane, as expected from the 2D character of the WO_3_·H_2_O structure. However, the W400 inhibited the Raman band at 948.3 cm^−1^ and two strong characteristic bands appeared at 812.13 cm^−1^ and 711.80 cm^−1^, indicating the stretching mode of the tungsten atom with a neighboring oxygen atom *ν*(O–W–O). The band at 276 cm^−1^ represents the bending vibration of the *δ*(O–W–O) mode. These three special vibration bands indicated the 3D network of the pure monoclinic WO_3_ structure. Interestingly, W200 also depicted the characteristics hydrate band at the same position of 948.3 cm^−1^, with a lower intensity as compared to W80. W200 showed a similar Raman band at 812 cm^−1^ due to the stretching mode of *ν*(O–W–O) as the monoclinic structure; the other band at 699 cm^−1^ shifted to the lower frequency region as compared to the band position of the pure monoclinic WO_3_ phase at 717 cm^−1^ for W400. The band at 699 cm^−1^ represents the characteristics peak of the hexagonal structure of WO_3_ (h-WO_3_).^41^ The intensity ratio of the W=O/W–O–W Raman band showed the crystallinity of W80, W200 and W400. Thus, the higher intensity of the W=O band and lower intensity of the W–O–W band illustrated the lower crystallinity in WO_3_.^42^ The estimated intensity ratios of W=O/W–O–W for W80, W200, and W400 were 1.19, 0.345, and 0.048, which indicated the systematic increase in the crystallinity with annealing temperature. Fig. 1(c) shows a schematic representation of the phase transition from the 2D layered orthorhombic WO_3_·H_2_O to the anhydrous 3D monoclinic WO_3_ structure as a function of the annealed temperature. The FTIR spectra elucidated the various functional groups present on the surface of W80, W200, and W400 as shown in Fig. 2(a). W80 showed the two prominent bands of the stretching and bending vibrations of *ν*(OH) and *δ*(OH) corresponding to the water molecules at 3397 cm^−1^ and 1604 cm^−1^, respectively. The peak at 947 cm^−1^ is attributed to a terminal oxygen atom (W=O) of the layered WO_3_·H_2_O structure. The band at 624 cm^−1^ is due to characteristic stretching vibrations of bridging *ν*(O–W–O) bonds. However, the W400 inhibits all the characteristic bands such as the terminal oxygen atom (W=O) at 947 cm^−1^, stretching *ν*(OH) at 3397 cm^−1^, and the bending vibration *δ*(OH) at 1604 cm^−1^, except the band of the bridging *ν*(O–W–O) network at 624 cm^−1^. Interestingly, at the intermediate annealing temperature of 200 °C, W200 exhibited the major characteristic band of the structural water of the terminal W=O at 1001 cm^−1^, which shifted to higher frequency. Also, W200 noticeably showed the bending *δ*(OH) vibration and bridging *ν*(O–W–O) bond at the same position as W80. The FTIR spectra of W80, W200, and W400 are in good agreement with the XRD patterns and Raman spectra. TGA analysis as shown in Fig. 2(b) exhibited the one-step total weight loss of 5% in the temperature range of 100 °C to 300 °C due to the confined structural water between the WO_3_·H_2_O layers. Therefore, it is important to investigate the electrochemical properties of WO_3_ nanoplates within the temperature range of major weight loss.^43^

**Fig. 3:**
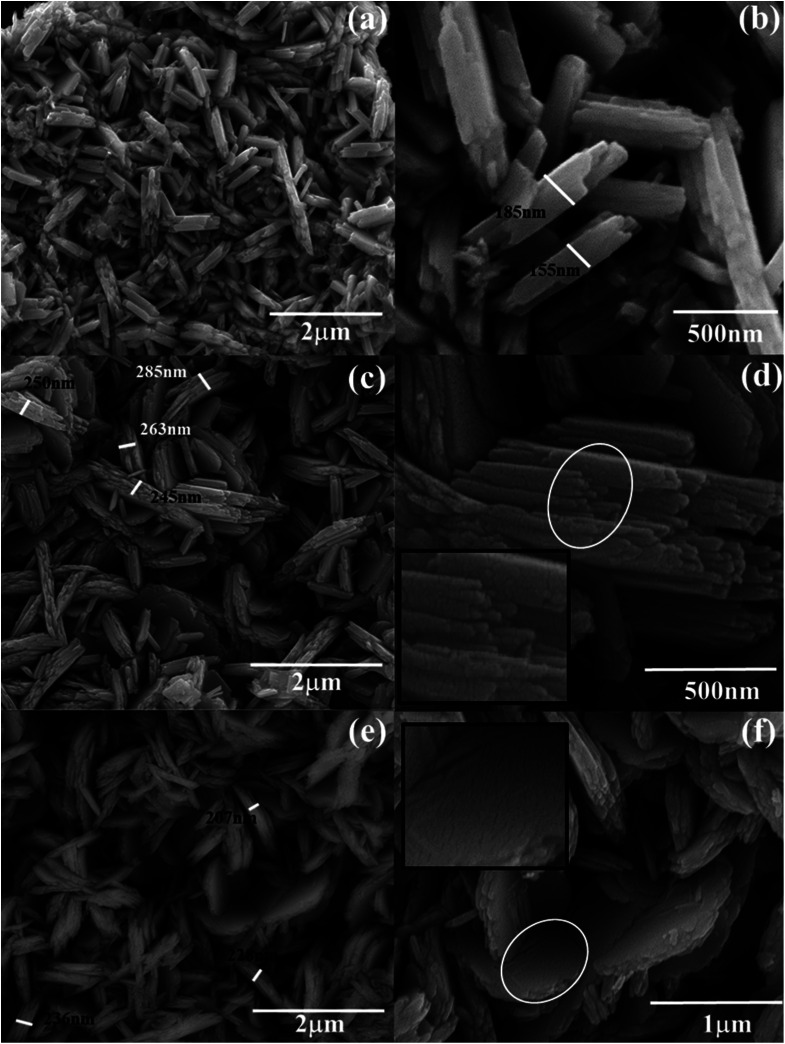
FESEM: (a and b) low and high-resolution images, respectively, of W80; (c and d) low and high-resolution images, respectively, of W200. The inset of (d) is the enlarged view of the selected circular region. (e and f) Low and high-resolution images, respectively, of W400. The inset of (f) is the enlarged view of the selected circular region.

**Fig. 4:**
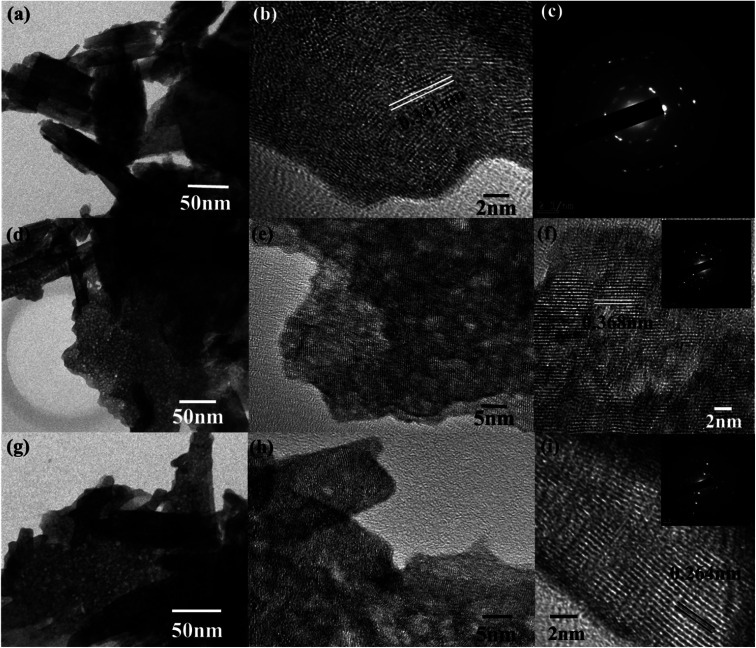
(a) Low-resolution TEM images of W80. (b and c) The HRTEM and SAED pattern, respectively, of W80. (d) Low-resolution TEM images of W200. (e and f) The HRTEM images of W200. The inset of (f) shows the SAED pattern of W200. (g) The low-resolution TEM image of W400. (h and i) The HRTEM images of W400. The inset of (i) shows the SAED pattern of W400.

**Fig. 5:**
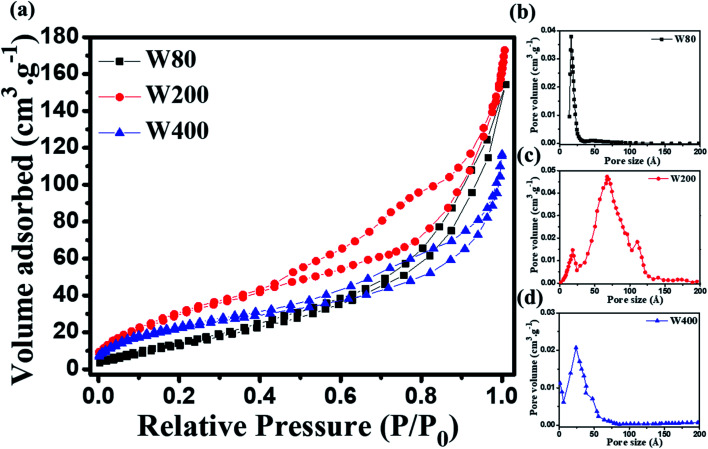
(a) Nitrogen adsorption–desorption isotherms of W80, W200 and W400. (b–d) Pore size distribution curves of W80, W200 and W400 acquired by the BJH method.

The morphological analyses of W80, W200, and W400 were performed by FESEM and HRTEM as shown in Fig. 3 and 4, respectively. The low-resolution FESEM images (Fig. 3(a)) of W80 depict the nanoplate-like structures. However, the high-resolution image (Fig. 3(b)) shows that the thickness of the plates varied from 150–200 nm and they were composed of 4–5 nanosheets, each with thickness ranging from 40 to 80 nm. The thickness of the W200 sample increased (Fig. 3(c)) to 250–300 nm without any morphology change but the systematic agglomeration of nanosheets along the vertical direction forming nanoplates was quite noticeable. The high-resolution images of W200 (Fig. 3(d)) indicate the porous nature of the nanoplates, which is an obvious result of the evaporation of the structural water.^30^ The inset of Fig. 3(d) shows a more clear view of the granular structure due to the porous network. The low-resolution image of W400 shown in Fig. 3(e) indicates the shrinking of the nanoplate thickness from 200 nm to 250 nm and the high-resolution image (Fig. 3(f)) confirmed a similar morphology. The inset of Fig. 3(f) illustrates the surface of the nanoplate. In Fig. 4(a), the low-resolution TEM image of W80 does not reveal any porous character. The HRTEM image (Fig. 4(b)) exhibits the interplanar distance of 0.34 nm, which corresponds to the *d*-spacing of the (111) plane. The SAED pattern in Fig. 4(c) confirmed the high crystallinity of W80. The low-resolution TEM image of W200 in Fig. 4(d) indicates a porous network on the surface, highlighting the granular nature and black dots in the image representing voids. Further, the HRTEM image (Fig. 4(e)) revealed the well-resolved interplanar lines and the average pore size was in the range of 3 to 5 nm. Fig. 4(f) depicts the well-aligned lattice of the interplanar distance of 0.368 nm in the direction of the (002) plane. The inset of Fig. 4(f) shows the SAED pattern illustrating the bright diffraction spots, indicating the single-crystalline nature of W200. Moreover, the low-resolution TEM image of W400 (Fig. 4(g)) illustrates a similar porous nature to W200; however, the HRTEM image (Fig. 4(h)) demonstrates the highly ordered lattice fringes. The interplanar distance (Fig. 4(i)) of W400 decreased to 0.263 nm in the direction of the plane (202). The inset of Fig. 4(i) shows the SAED pattern confirming the single-crystalline nature. The surface area and pore size distribution of W80, W200 and W400 were investigated by the Brunauer–Emmett–Teller (BET) and Barrett–Joyner–Halenda (BJH) methods, respectively. Fig. 5(a) depicts the comparative nitrogen adsorption–desorption isotherms of all three samples. W200 and W400 showed the Langmuir type II isotherm and H3 hysteresis loop, which revealed the existence of the mesoporous structure. W80 exhibited the type III isotherm and the same H3 hysteresis loop. The obtained BET surface areas of W80, W200 and W400 were 68.8 m^2^ g^−1^, 124.7 m^2^ g^−1^ and 57.4 m^2^ g^−1^, respectively. Fig. 5(b–d) shows the pore size distribution of W80, W200 and W400. The highest surface area, pore volume and pore size of W200 (values listed in Table 1) compared to W80 and W400 ensure more surface active sites for fast redox reactions, which is the most crucial property of electrode materials for electrochemical energy storage.^44–46^

**Table 1:** Morphological and electrochemical properties of the three samples prepared at post-annealing temperatures^a^

Sample	C.S	*D*, nm	*S*_a_, m^2^ g^−1^	*P*_s_, nm	*P*_v_, cm^3^ g^−1^	S.C (F g^−1^)
W80	Orthorhombic	36.4	68.8	1.9	0.13	361
W200	Mixed-phase	11.3	124.7	7.3	0.24	606
W400	Monoclinic	27.2	57.4	2.7	0.17	302

a C.S: crystal structure, *D*: crystalline size, *S*_a_: surface area, *P*_s_: pore size, *P*_v_: pore volume, S.C: specific capacitance.

### 3.2 Electrochemical characterizations

**Fig. 6:**
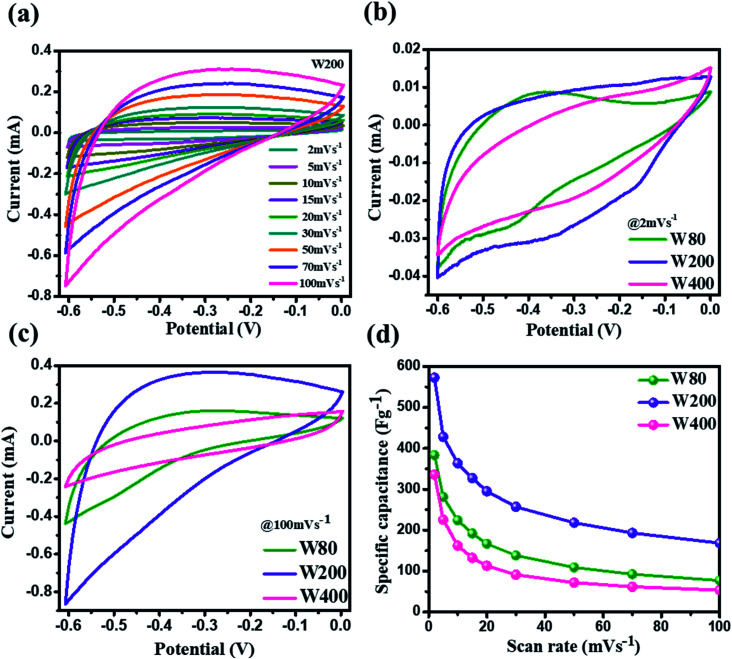
(a) Cyclic voltammetry curves of W200 at various scan rates. (b) The comparative cyclic voltammetry curves of W80, W200, and W400 at the scan rate of 2 mV s^−1^. (c) The comparative cyclic voltammetry curve of W80, W200, and W400 at the scan rate of 100 mV s^−1^. (d) The specific capacitance *vs.* scan rates of W80, W200, and W400.

The electrochemical charge storage properties of W80, W200, and W400 were performed by cyclic voltammetry (CV) and galvanostatic charge–discharge (GCD) in a potential window between 0 V to −0.6 V using aqueous 1 M H_2_SO_4_ as the electrolyte. Fig. 6(a) illustrates the CV curve of the W200 electrode at various scan rates from 2 mV s^−1^ to 100 mV s^−1^ and similarly, Fig. S3(a) and (b)† show CV curves of W80 and W400, respectively. It is noted that the anodic and cathode current increased as the scan rate increased from 2 mV s^−1^ to 100 mV s^−1^. The comparative CV curve (Fig. 6(b)) at a lower scan rate of 2 mV s^−1^ demonstrates that electrode W200 retains the greater area under the CV curve as compared to electrode W80 and W400. The CV curve shows the quasi-rectangular nature due to the pseudocapacitive charge storage. The W80 electrode emphasizes the prominent reversible redox reaction, *i.e.*, cathodic and anodic peaks arising from the reduction of W^6+^ to W^5+^ at the potential of −0.4 V to −0.5 V and −0.35 V to −0.45 V, respectively, which has shown by reaction (b):^47^

b WO_3_ + *x*H^+^ + *x*e^−^ ↔ H*x*WO_3_

**Fig. 7:**
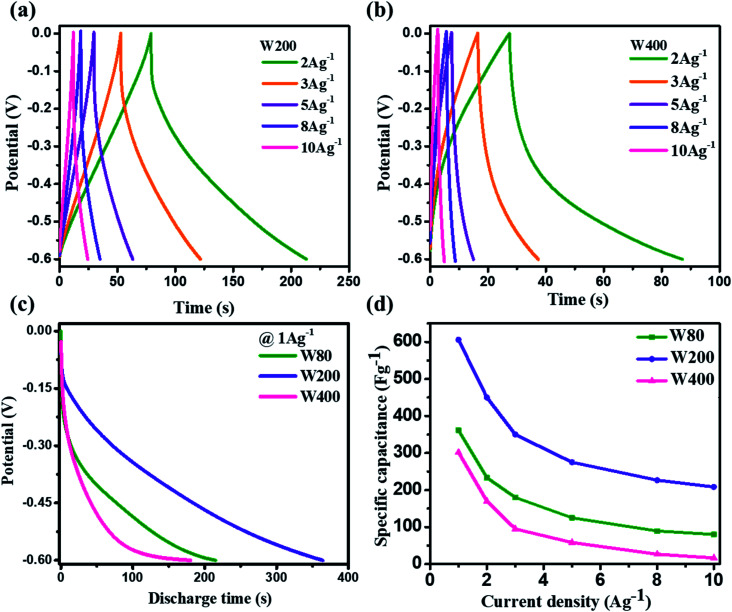
(a and b) Galvanostatic charge–discharge curves of the W200 and W400 at various current densities. (c) The comparative galvanostatic discharge curves of W80, W200, and W400 at a current density of 1 A g^−1^. (d) Specific capacitances *vs.* current densities for W80, W200 and W400.

Electrode W200 demonstrated an irreversible faradaic reaction accomplished by the two-step intercalation of a proton into the porous network of the mixed-phase at a potential in the ranges of −0.1 V to −0.2 V, and −0.35 to −0.45 V. Electrode W400 showed the smallest area under the CV curve, with no indication of a redox reaction, and thus had the smallest capacitance as compared to electrodes W200 and W80. However, at a high scan-rate of 100 mV s^−1^ (Fig. 6(c)), electrodes W80 and W200 did not show any redox peaks, due to the fast proton kinetics under the high electric field, whereas the major charge storage contribution arose from the proton adsorption/desorption at the interface of the electrodes. Fig. 6(d) exhibits the highest specific capacitance of electrode W200 (572 F g^−1^) as compared to electrode W80 (386 F g^−1^) and W400 (336 F g^−1^) at a scan-rate of 2 mV s^−1^. Fig. 7(a and b) represent the GCD curves of electrodes W200 and W400, respectively, and Fig. S4† represents W80 at various current densities from 2 to 10 A g^−1^. The GCD curves showed the distorted triangular curve representing the pseudocapacitive charge storage as indicated by the CV curves. Fig. 7(c) shows the highest discharge time of electrode W200 as compared to W80 and W400 at a current density of 1 A g^−1^. The specific capacitance at various current densities (Fig. 7(d)) shows the highest specific capacitance of 606 F g^−1^ of the electrode W200 as compared to electrode W80 (361 F g^−1^) and W400 (302 F g^−1^) at a current density of 1 A g^−1^. Electrode W200 also revealed superior specific capacitance over other WO_3_ nanostructures reported earlier in the literature (Table 2). However, at a high current density of 10 A g^−1^, electrode W200 maintained the specific capacitance of 208 F g^−1^, which is higher as compared to electrodes W80 (80 F g^−1^) and W400 (18 F g^−1^). Electrode W200 also demonstrated an excellent capability of 76%, which is three-fold higher than electrode W400 (26%) and better than W80 (64%) between the current densities of 3 and 10 A g^−1^.

**Table 2:** Comparison table of various WO_3_ crystal structures, morphologies and their specific capacitances

Material	Method	Morphology	Crystal structure	Specific capacitance (F g^−1^)	Ref.
WO_3_	Hexagonal	Cubic plates	Monoclinic	368	24
h-WO_3_	Hydrothermal	Nano fiber	Hexagonal	436	21
h-WO_3_·*n*H_2_O	Hydrothermal	Nanorod	Hexagonal	391	28
h-WO_3_	Hydrothermal	Nanopillar	Hexagonal	421	32
WO_3_	Hydrothermal	Nano flakes	Hexagonal	538	52
WO_3_	Hydrothermal	Nanorod	Hexagonal	538	53
WO_3_	Hydrothermal	Nanorod	Hexagonal	319	54
WO_3_	Hydrothermal	Micro-rod	NA	94.2	56
WO_3_–WO_3_·0.5H_2_O	Microwave-assisted hydrothermal	Nanorod	Hexagonal + cubic	290	57
WO_3_ nanoplates	Wet-chemical	Mesoporous nanoplates	Orthorhombic + hexagonal + monoclinic	606	This work

**Fig. 8:**
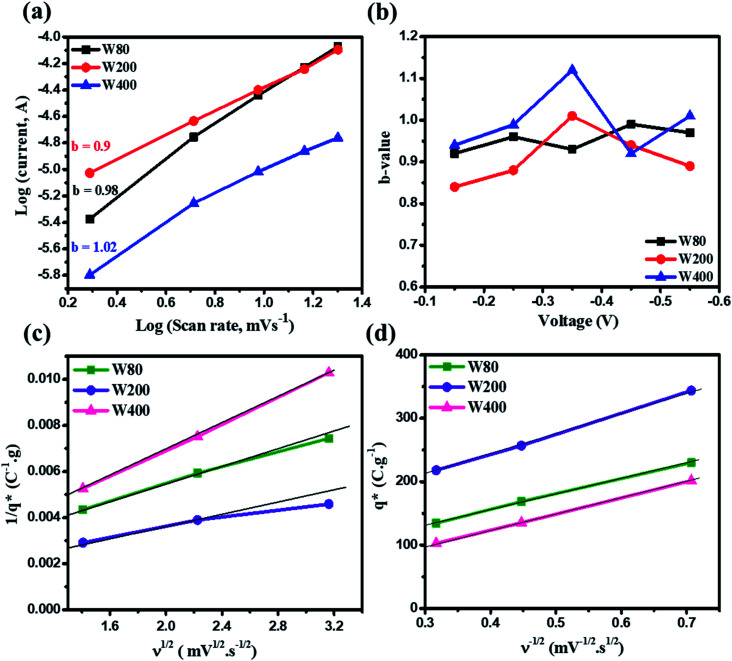
(a) Plots of log(scan rate) *vs.* log(current) for estimating the *b*-values of samples W80, W200 and W400. (b) Plots of *b*-values *vs.* voltage for samples W80, W200 and W400. Trasatti method: (c) plot of 1/q* *vs. ν*^1/2^, (d) plot of q* *vs. ν*^−1/2^ for samples W80, W200 and W400.

The relationship between the scan rate (*ν*)-dependent current contributions in the CV curve is given by eqn (2);

2 *I* = *ν*^*b*^

where *I* and *ν* are the current and scan rate, *b* is a constant value that can either be 0.5 or 1.^48–50^ The *b*-value of unity in eqn (2) reveals the direct relationship between current and scan rate, which highlight the dominant capacitive charge storage mechanism. However, a *b*-value equal to 0.5 indicates that the charge storage in the electrode materials is dominated by the ionic diffusion process. The *b*-value was obtained from the slope of the plot of the log(current) *vs.* log(*ν*) with the scan rate of 2–20 mV s^−1^. Fig. 8(a) shows the anodic peak *b*-value of 0.98, 0.9 and 1.02 of electrodes W80, W200 and W400, respectively. The comparatively lower *b*-value of electrode W200 showed significant proton insertion into the electrode materials attributed to the mesoporous nature and high surface area. The voltage-dependent *b*-values (Fig. 8(b)) of W80, W200 and W400 demonstrate the comparatively lower *b*-value of electrode W200 as compared to W80 and W400. Moreover, the quantitative estimation of the charge stored at the outer and inner surface of electrodes W80, W200 and W400 was done by using the Trasatti method.^51^ This method is based on the voltammetry charge (q*) as a function of the scan-rate (mV s^−1^). At a high scan-rate, the insertion of a proton into the inner surface becomes more difficult. Hence, at the scan rate *ν* → ∞, all the inner surfaces of the electrode are excluded and q* tends toward the outer surface charge 
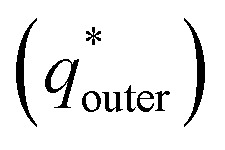
. However, as the scan-rate tends to zero (*ν* → 0), all inner surfaces are fully accessible and q* tends toward the total charge 
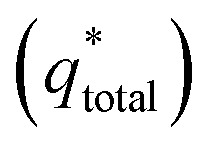
. The 
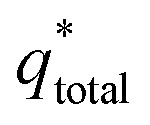
 of the electrode can be obtained by the plot of 1/q* *vs. ν*^1/2^ (Fig. 8(c)), where the intercept of the fitted straight-line gives the total charge stored in the electrode. The charges stored in the W80, W200, and W400 electrodes were 243 C g^−1^, 384 C g^−1^, and 169 C g^−1^, respectively. Similarly, 
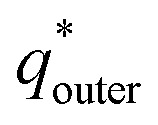
 of the electrode was obtained from the intercept of plots of q* *vs. ν*^−1/2^ (Fig. 8(d)). The charges stored on the surface of electrodes W80, W200, and W400 were 131 C g^−1^, 215 C g^−1^, and 95 C g^−1^, respectively. The charge stored at the inner surfaces such as pores and cracks is the difference between charges at the outer surface and the total surface. The calculated charge stored inside electrodes W80, W200, and W400 were 112 C g^−1^, 169 C g^−1^, and 98 C g^−1^, respectively.

**Fig. 9:**
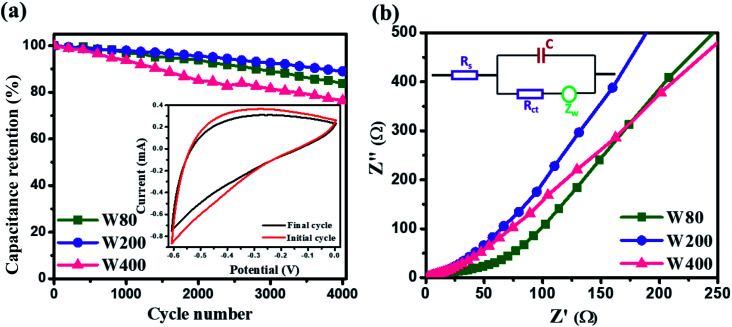
(a) Cyclic stability test; the inset shows the CV curve before and after 4000 cycles. (b) Nyquist plot of W80, W200 and W400 and the inset figure shows the equivalent circuit diagram.

The cyclic stability test was performed at a high scan-rate of 100 mV s^−1^ (Fig. 9(a)), which demonstrated the higher capacitance retention of electrode W200 (89%) than W80 (83%) and W400 (76%) after 4000 cycles. The inset of Fig. 9(a) shows the CV curve at 100 mV s^−1^, before and after 4000 cycles. Fig. 9(b) shows the electrochemical impedance spectroscopy of electrodes W80, W200, and W400 in the frequency region of 0.01 Hz to 100 kHz. The Nyquist plot of electrode W80 shows the semi-circle at the high-frequency region, highlighting the charge transfer resistance (*R*_ct_) of 40 Ω across the interface of the electrode and electrolyte.^52^ Electrodes W200 and W400 did not exhibit the charge transfer resistance, revealing the higher conductivity. The *x*-axial intercept at the high-frequency region represents the equivalent series resistance (*R*_ESR_) corresponding to the resistance of the current collector-electrode and the ohmic resistance of the electrolyte/electrode interface.^53^ The obtained *R*_ESR_ values were 3.11 Ω, 1.45 Ω and 1.51 Ω for electrodes W80, W200 and W400, respectively. Thus, the lower value of *R*_ESR_ suggests that the W200 structure enabled enhanced proton transport into the internal surface of the electrode. Furthermore, the obtained Warburg impedance (*Z*_w_) of electrodes W80, W200, and W400 were 83 Ω, 26 Ω, and 35 Ω, respectively. The lower *Z*_w_ of electrode W200 revealed the fast proton diffusion and transport throughout the electrode.^54,55^ The inset of Fig. 9(b) shows the equivalent circuit diagram comprising the components of *C*, *Z*_w_, *R*_ct_ and *R*_ESR_. Thus, the W200 electrode facilitating the mixed crystal phase, porous network and agglomerated nanoplates morphology achieved by annealing at 200 °C provides highly suitable electrochemical features as an electrode material for future supercapacitor application.

## 4. Conclusion

The phase transition and the porous network in WO_3_ nanoplates were achieved by a post-annealing method for supercapacitor applications. Annealing at 80 °C, 200 °C, and 400 °C demonstrated the phase transition from the orthorhombic structure of the hydrate WO_3_·H_2_O (W80) to the mixed-phase (W200) and finally the pure monoclinic crystal structure of WO_3_ nanoplates (W400), respectively, owing to the escaping of the structural hydrate without disturbing the nanoplate morphology. The phase transitions and the improved degrees of crystallinity at 80, 200 and 400 °C were confirmed by XRD and Raman analysis. The W200 electrode demonstrated superior specific capacitance of 606 F g^−1^ as compared to the W80 electrode (346 F g^−1^) and it was two-fold higher than the W400 electrode (302 F g^−1^). The W200 electrode also exhibited an excellent rate capability of 76%, about three-fold higher than electrode W400 (26%). Moreover, the W200 electrode illustrated a better capacitance retention of 89% as compared to electrodes W80 (86%) and W400 (76%). Thus, the W200 electrode materials with mixed crystal phase, porous network, high surface area and agglomerated nanoplate architecture favor extremely suitable electrochemical qualities as potential electrode material for supercapacitors.

## Conflicts of interest

There are no conflicts to declare.

## Supplementary Material

NA-002-D0NA00423E-s001
