# Supplementary material for: Phase transformation in tungsten oxide nanoplates as a function of post-annealing temperature and its electrochemical influence on energy storage
Source: Nanoscale Adv. 2020 Aug 6;2(10):4689–701. doi: 10.1039/d0na00423e (PMC9416815; doi:10.1039/d0na00423e)
Supplement: NA-002-D0NA00423E-s001 [file NA-002-D0NA00423E-s001.pdf]

Electronic Supplementary Material (ESI) for Nanoscale Advances.
This journal is © The Royal Society of Chemistry 2020

Supporting Information

Phase transformation in tungsten oxide nanoplates as a function of post-annealing temperature and its electrochemical influence on energy storage

Shobhnath P. Gupta, [a] **Harishchandra H. Nishad,**[a] **Sanjay D. Chakane,**[b] **Suresh W. Gosavi,**[c] **Dattatray J. Late,**[d] **Pravin S. Walke** [a,*]

[a] National Centre for Nanoscience's and Nanotechnology, University of Mumbai, Mumbai-400098, India.

[b] Department of Physics, Arts, Science and Commerce College, Indapur, Pune-413106, Affiliated to Savitribai Phule Pune University, Pune-411007, India.

[c] Department of Physics, Savitribai Phule Pune University, Pune-411007, India.

[d] Centre for Nanoscience and Nanotechnology, Amity University, Mumbai-410206, India.

*Corresponding author: Tel: +918380832183, E-mail: pravin.w@nano.mu.ac.in; shivshripsw@gmail.com **(Pravin Walke)**

Contains:

Numerical methods.

Figures S1 to S4.

A. Numerical Methods

1. Specific capacitance calculated from CV curve as given as:

$$C = \frac{\int_{V_a}^{V_b} IdV}{mV(V_b - V_a)} \tag{1}$$

Where, *I.dV* is total area under the CV curve at given potential range, *m* is mass of material, *V* is total scan range, V_a and V_b are initial and final voltage of given potential range in CV curve respectively.

2. Specific capacitance from charge discharge curve is given as:

$$C = \frac{I\Delta t}{m\Delta V} \tag{2}$$

where *I/m* is applied current density, Δt is discharge time, ΔV is total applied voltage range.

3. The trasatti equation as follows:

$$1/q^*(\nu) = 1/q^*_{total} + a\nu^{1/2} \tag{3}$$

Where, q* is the maximum charge stored, ν is scan rate, *a* is constant, q^*_{total} is total charges stored at inner and outer surface of electrode.

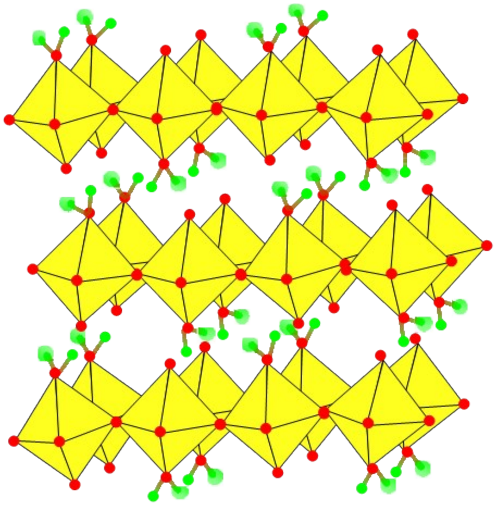

Fig. S1. Crystal structure of 2D layered $WO_3.H_2O$ (W80).

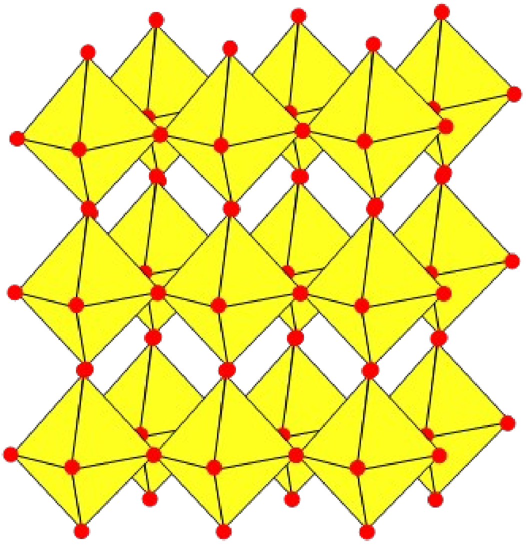

Fig. S2. Crystal structure of 3D monoclinic WO_3 (W400).

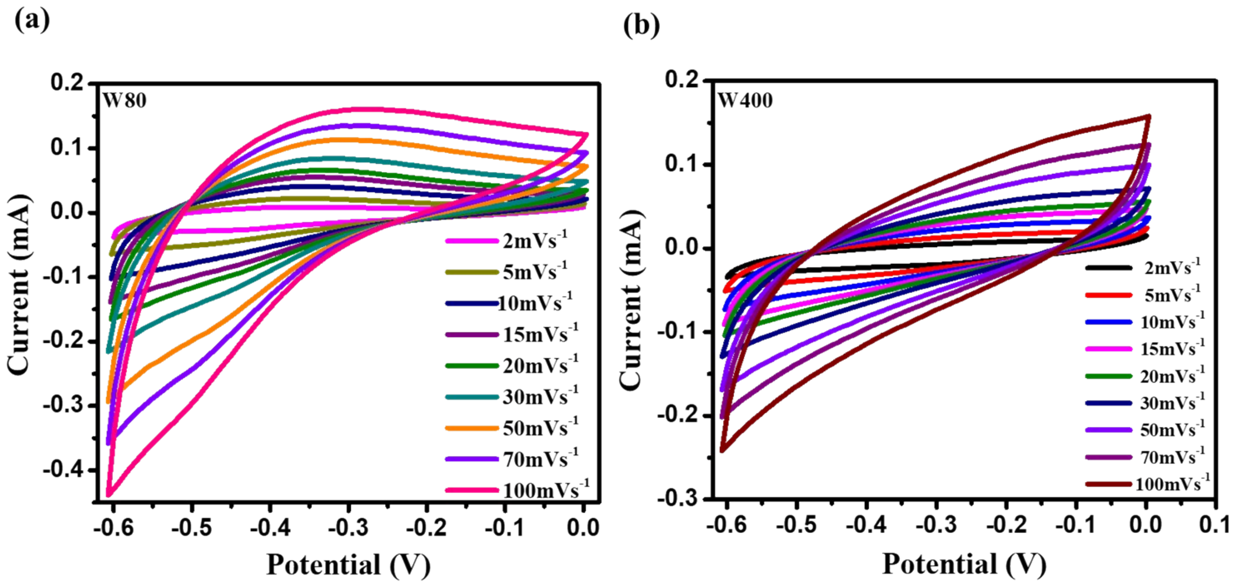

Fig. S3. Cyclic voltammetry curves of (a) W80, (b) W400 at various scan rates.

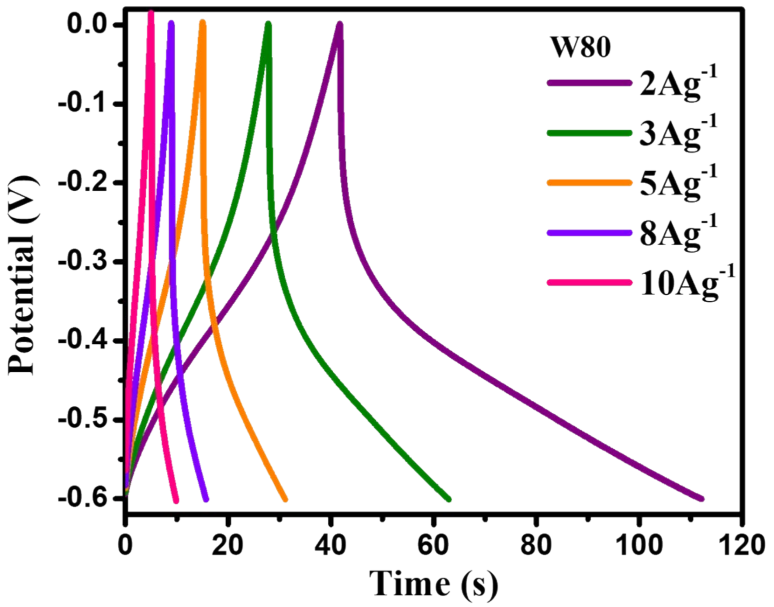

Fig. S4. Galvanostatics charge-discharge curves of W80 at various current densities.
